# Retraction: Clinical implications of first-trimester ultrasound dating in singleton pregnancies obtained through *in vitro* fertilization

**DOI:** 10.1371/journal.pone.0338856

**Published:** 2025-12-18

**Authors:** The PLOS One Editors

The *PLOS One* Editors retract this article [1] because it was identified as one of a series of submissions for which we have concerns about compromised peer review.

ES, CD, and VA agreed with the retraction. AMCR, MR, MGP, NP, and SB responded but expressed neither agreement nor disagreement with the editorial decision. GF either did not respond directly or could not be reached.
